# Targeting neutrophils: Mechanism and advances in cancer therapy

**DOI:** 10.1002/ctm2.1599

**Published:** 2024-03-07

**Authors:** Shuaixi Yang, Jiachi Jia, Fuqi Wang, Yuhang Wang, Yingshuai Fang, Yabing Yang, Quanbo Zhou, Weitang Yuan, Zhilei Bian

**Affiliations:** ^1^ Department of Colorectal Surgery The First Affiliated Hospital of Zhengzhou University Zhengzhou University Zhengzhou China; ^2^ Department of Hematology The First Affiliated Hospital of Zhengzhou University Zhengzhou University Zhengzhou China

**Keywords:** NETs, neutrophils, targeting therapy, tumour microenvironment

## Abstract

**Background:**

Cancer is a thorny problem which cannot be conquered by mankind at present and recent researchers have put their focus on tumor microenviroment. Neutrophils, the prominent leukocytes in peripheral blood that accumulate in tumours, serves as frontline cells in response to tumour progression owing to the rapid development of micro biotechnology. Hence, targeted therapy with these neutrophils has made targeting treatment a promising field in cancer therapy.

**Main body:**

We broadly summarise some studies on the phenotypes and functions of tumour‐associated neutrophils as well as the unique web‐like products of neutrophils that play a role in cancer progression—neutrophil extracellular traps—and the interactions between neutrophils and the tumour microenvironment. Moreover, several targeted neutrophils therapeutic studies have made some progress and provided potential strategies for the treatment of cancer.

**Conclusion:**

This review aims to offer a holistic perspective on therapeutic interventions targeting neutrophils to further inspire more researches on cancer therapies.

## Graphical abstract

Our discussion predominantly centred on the distinctive role of neutrophils within the tumour microenvironment and the prospect of devising targeted therapies. Neutrophil extracellular traps play a pivotal role in contemporary cancer research which also become a subject extensively covered in our essay. At last, we provided a summary of ongoing therapies aimed at targeting neutrophils and illuminated potential therapeutic targets.

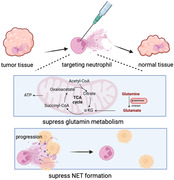

## 1 INTRODUCTION

In humans, neutrophils stand as the most plentiful immune cells and account for 50%–70% of all leukocytes.^1^ Its pathogenic roles are related to chronic inflammation and autoimmune diseases.^2^ Nevertheless, owing to their incapacity for proliferation post‐maturation and their limited half‐life, investigations into neutrophils within heterogeneous tumours have been notably absent in recent decades. According to the latest findings, neutrophils have attracted increasing attention in tumour‐relevant studies, conventional views on neutrophils have changed due to the new advances in biotechnology, such as high‐dimensional transcriptomic and epigenomic approaches.^3^ Research on neutrophils has evolved from focusing on the quantity and function of macroscopic to microscopic phenotypes and genes.

The reassessment of neutrophils led to the conclusion that neutrophils in tumours are classified into different subsets that have been polarised into antitumour and protumour phenotypes, known as N1 and N2, which could have contrary effects on every stage of tumours, including initiation, proliferation, metastasis and immunosuppression.^4^, ^5^, ^6^ However, it is worth noting that the heterogeneity of tumour‐associated neutrophils (TANs) is far beyond the simple classification in which more than nine different subsets have already been reported.^7^ New clinical studies investigating the role of neutrophils have brought targeting neutrophil therapy into the spotlight.^8^, ^9^ The newest approaches for targeting neutrophils are based on the different subgroups of tumours that exert various functions. That is, they act by suppressing protumour subsets and promoting antitumour subsets. In addition to neutrophils, neutrophils‐related components have also attracted increased amounts of attention. Neutrophil extracellular traps (NETs), a specific reticulated product of neutrophils, are in the focus of cancer therapy because they function as the forefront in medical intervention and play a prominent role in the establishment of the premetastatic niche. Crosstalk between neutrophils and other immune cells also sheds new light on cancer therapy.

In this brief review, we summarise some of relevant findings about neutrophils in tumours. We will review the new therapeutic targets identified by the latest findings concerning TANs.

## 2 ORIGIN AND PHENOTYPE

**FIGURE 1:**
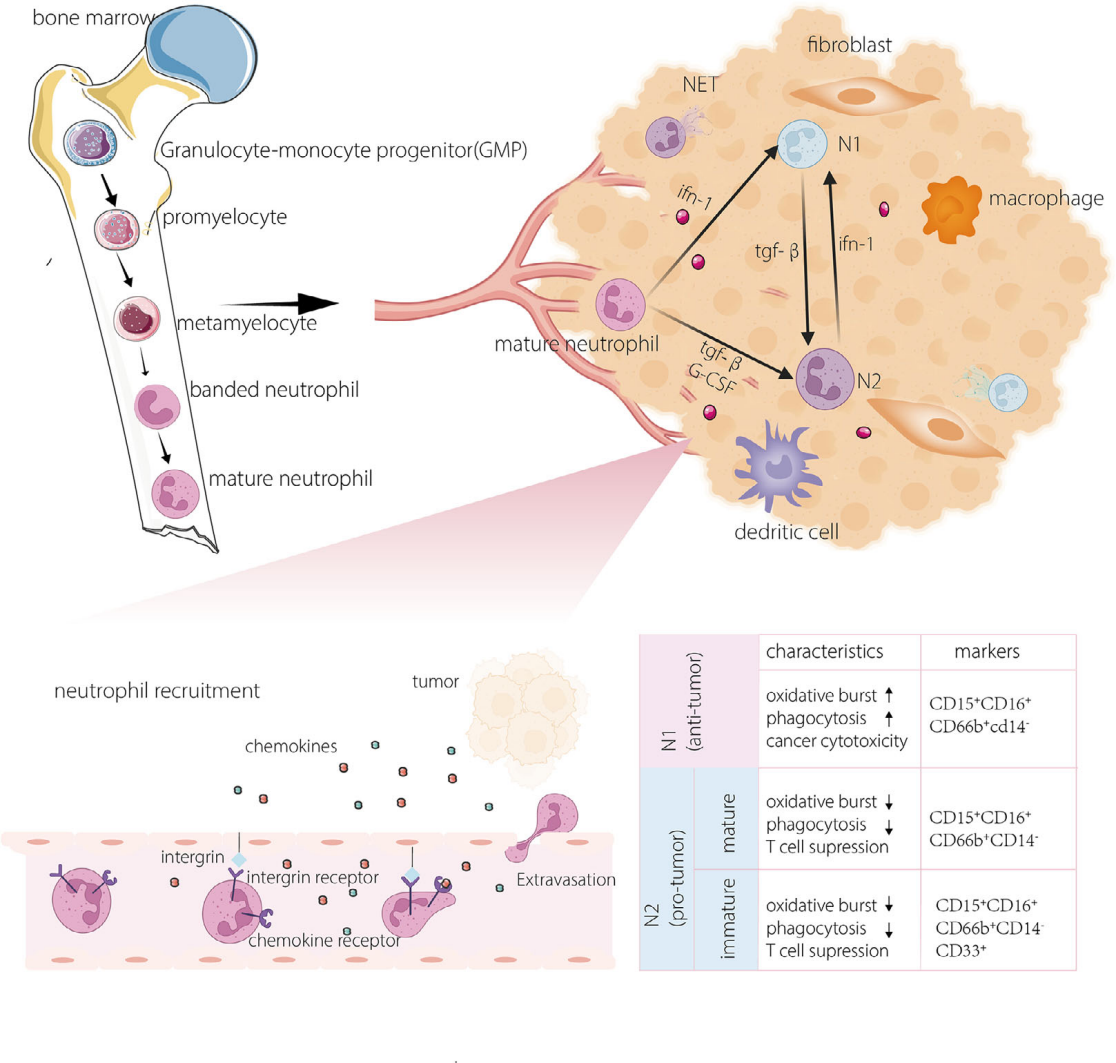
The origin, activation and phenotypical differences of neutrophils. Neutrophils are usually generated from myeloid stem cells in the bone marrow and go through a certain stage of development; these cells encompass myoblasts, neutrophil progenitors, immature neutrophils and mature neutrophils. Neutrophil recruitment, phenotypic plasticity and function are largely dependent on certain cytokines in related environments.

The origin, recruitment and phenotypic changes (switching between antitumour and protumour) of neutrophils in tumour tissues have been associated with certain clinical outcomes, and the specific role and pathogenesis of TANs have been well detected in recent studies. Under normal physiological circumstances, neutrophils are continually produced and housed in the bone marrow.^10^ The haematopoietic cords, situated within the bone marrow, serve as the source of neutrophils, with common myeloid progenitor cells representing the genuine wellspring of neutrophils within the venous sinuses.^11^, ^12^ With the help of transcription factors such as CCAAT‐enhancer binding protein(CEPB/α）, AML‐1 (acute myeloid leukemia 1)and proteins such as colony‐stimulating factors,^13^, ^14^ neutrophils can be produced without any complications. Due to the diversity of transcription factors and certain proteins, TANs can result in different phenotypes. Many studies on the differentiation of neutrophils have divide them into various subsets based on different criteria. Here, we summarise the most common classification results according to phenotypic and functional differences: the N1, or antitumour neutrophils, and the N2, or protumour neutrophils.^5^ N1 and N2 are the two most extensively studied cell populations in terms of their phenotype and function. Interferon type 1 (IFN‐1) is the major cause of neutrophils polarisation into the N1, which can increase adhesion, transmigration, phagocytosis, oxidative bursts, deregulation and NETs.^5^ Otherwise, in the presence of transforming growth factor‐β (TGF‐β), neutrophil differentiation progress towards the N2, which modulates the immune system.^15^ The switch between N1 and N2 may also suggest that IFN‐1 and TGF‐β may have an antagonistic signalling pathways^16^ (Figure 1). Notably, there are distinct differences between immature N2s and mature N2s as they differ under some pathological conditions.^17^

N1 cells, which are antitumour neutrophils, are characterised by cancer cells cytotoxicity mediated by reactive oxygen species (ROS) production, which is found to activate TRMP2, an H_2_O_2_‐dependent Ca^2+^ channel, ultimately leading to the lethal influx of calcium ions in tumour cells.^18^ Recent studies have unveiled other mechanisms of suppressing cancer cells^19^ as RNS secreted by N1s are absorbed by tumour cells and alter the expression of cancer‐related genes.^20^ On the contrary, N2 exhibits the ability to advance tumour progression through the generation of a significant array of enzymes, encompassing myeloperoxidase, neutrophil elastase (NE) and matrix metalloproteinases. These enzymes can remodel dense extracellular matrix (ECM) to promote angiogenesis and cancer migration.^21^

Certain neutrophils can be involved in cancer. Chemotaxis, widely recognised as the directed cellular movement in response to specific chemical signals, plays an important role in guiding neutrophils recruitment to tumour tissues.^22^, ^23^ Chemokines, discharged by tumour cells, can traverse the capillary endothelium and become entrapped by glycosaminoglycans or heparan sulphate on the inner membrane of endothelial cells, where they are then acknowledged by receptors on the surface of neutrophils.^22^ The recruitment of neutrophils is a significant step in neutrophil polarisation since the diversity of neutrophils is largely affected by the interactions between neutrophils and the tumour microenvironment (TME). Based on pertinent research, neutrophils possessing an inherent antitumour capability are enlisted into the TME and subsequently undergo a transformation into a protumour phenotype. The reprogramming of neutrophils also provides new insights into neutrophil communication with the TME.

However, importantly, the study of neutrophils still has many shortcomings due to their short lifespan and the incomplete understanding of marker types. Research on neutrophils with phenotypes other than N1 and N2 is still in its early stages.^24^

## 3 NET AND TUMOUR EVOLUTION

Due to the heterogeneity of cancer cells and the intricate elements within the TME, extensive research on neutrophils is imperative.^25^, ^26^ With the development of single cell RNA sequencing(scRNA‐seq), we identified distinct phenotypes and maturation states by observing differences in the expression of enriched genes—*sell^hi^ ngp^hi^*/*lst^hi^*, *sell^hi^ cxcl* ^10^.^7^, ^27^ The two types of neutrophils, known as N1 and the N2, perform diametrically opposite functions in tumours. NETs, which are formed by viable neutrophils containing mitochondrial DNA, were lately discovered to potentially serve as a bridge for interactions among neutrophils and neutrophils because they are unique meshworks to neutrophils.^28^ Aligned with the phenotypic characteristics of neutrophils, NETs have been recognised for their involvement in both anti‐inflammatory and proinflammatory processes.^26^

### 3.1 NET germination

**FIGURE 2:**
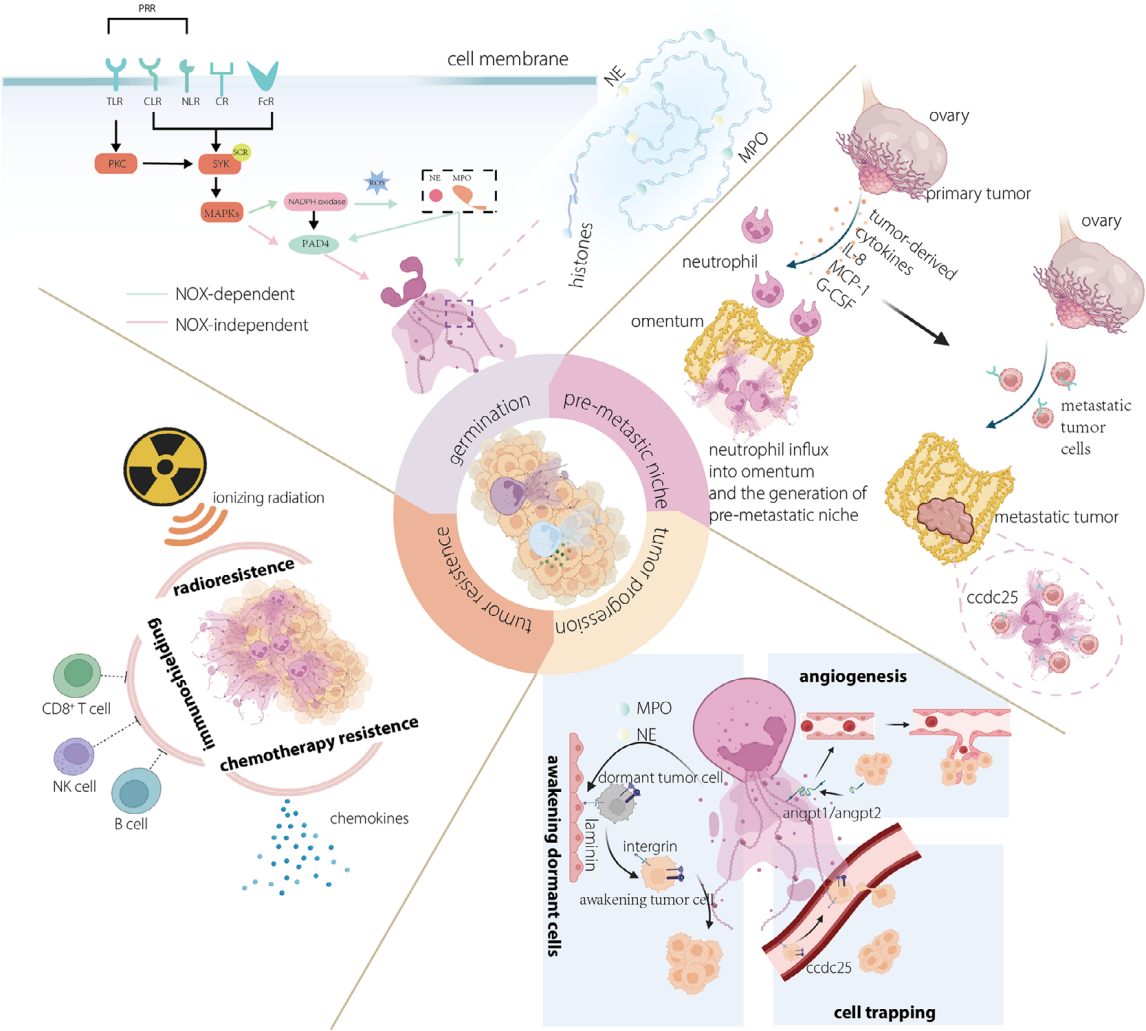
A brief summary of the detailed information on neutrophil extracellular traps (NETs). The molecular process underlying NET production can be delineated into two primary pathways: NOX‐dependent and NOX‐independent pathways. Upon pathological stimulation by tumours, intricate NET structures are formed, comprising chromatin along with specific microbicidal proteins, including neutrophil elastase (NE) and myeloperoxidase (MPO). The protumour aspects of NETs can be classified as tumour progression or tumour resistance. NETs possess the capability to rouse dormant tumour cells, ensnare them to facilitate metastasis and stimulate angiogenesis to nourish tumour tissues. When NETs overlay tumour cells, they act as a protective screen, shielding tumours from radioactivity, immune cells and chemokines for treatment.

NETs, intricate structures resembling webs and comprising cytosolic and microbicidal proteins, are assembled through the scaffolding of decondensed chromatin (Figure 2) and are initially recognised as a novel mechanism causing cellular harm in bacterial contexts.^29^, ^30^ The process of this neutrophil‐induced NETosis requires ROS and nicotinamide adenine dinucleotide phosphate(NADPH) oxidase after stimulation.^31^ Receptors such as pattern recognition receptors, complement receptors (CRs), Fc receptors and chemokine receptors (CXCRs) are to react to different stimuli,^29^, ^32^ but the signalling pathways implicated in these processes remain incompletely elucidated. Some signalling pathways are fully understood, such as the p38 mitogen‐activated protein kinase signalling pathway,^32^ and the FcγIII*b* crosslinking TAK‐1‐dependent mitogen‐activated extracellular signal‐regulated kinase/extracellular signal‐regulated (kiMEK/ERK) signalling pathway^33^, ^34^ (Figure 2). In this signalling pathway, the germination of NET is divided into two different methods: NADPH oxidase(NOX)‐dependent NETosis and NOX‐independent NETosis.^35^ Notably, some kinases, such as phosphoinositide 3‐kinase (PI3K), have not been classified into any signalling pathway.^29^ The subsequent product of the previously mentioned signalling pathways is ROS, which can activate peptidyl arginine deiminase 4 (PAD4), NE and myeloperoxidase (MPO).^36^ These modifications will utterly promote the unfolding of chromatin and the disappearance of membranous structures. NETs play key roles in infectious disease, chronic inflammation and tissue repair.^37^ It has been shown that NETs can form barriers to prevent lesions or separate necrotic areas from the healthy organs.^38^ The presence of NETs in tumour tissues has become an attractive area as biotechnology has progressed substantially, allowing researchers to further study the function of neutrophils in cancer.

### 3.2 NET and premetastatic niche

The premetastatic niche plays a crucial role in facilitating the colonisation of cancer cells in distant organs,^39^ and has also has attracted much attention in cancer‐targeting treatment.^40^, ^41^ Premetastatic niches, which are the foundation of tumour metastasis, are no longer considered to take place in one location. Circulating tumour cells are captured and vascular permeability is increased. NETs, key components the of premetastatic niche, can promote tumour cell intravasation and the establishment of metastasis.^42^ For example, Lee et al. described the premetastatic niche of ovarian formed by NETs in the omentum^43^ (Figure 2). Until recently, the precise mechanism responsible for the formation of the premetastatic niche had remained elusive. In recent in vitro studies concerning tumour metastasis in gastric cancer, researchers unveiled that the promotion of proliferation, invasion, migration and epithelial–mesenchymal transition (EMT) in gastric cancer cells induced by NETs relied on the activation of the TGF‐β signalling pathway.^44^

### 3.3 NET and tumour progression

Recent inquiries have elucidated a connection between NETs and the advancement of cancer^45^, ^46^ (Figure 2). Numerous studies have demonstrated that cancer cells can induce the generation of NETs through the secretion of chemokines and proteases.^25^ The potential underlying mechanism could entail the capability of NETs to undergo capture or the initiation of chemotaxis and adhesion facilitated by a DNA receptor that directly engages with NETs or integrins.^47^, ^48^ The vigorous growth and development of tumours make the tumour cells necessitate a sufficient provision of oxygen and nutrients, which are provided by vessels. Angiogenesis, defined as the process of neovascularisation, is enriched by the release of NETs induced by the recognition of angiopoietin 1/2 by Tie 2.^49^, ^50^ NETs may also directly facilitate the proliferation of tumour cells. For instance, Houghton et al. suggested that NETs formed in Lewis lung cancer may directly promote tumour growth, as these tumour cells grow more slowly in PAD4‐deficient mice than in control mice.^51^

### 3.4 NET and tumour resistance

Drug‐resistant cancer is an imperative health concern of worldwide interest and this overarching significance is mainly what makes carcinoma an incurable disease. Research on resistance has never failed to attract scholars’ attention. The presence of NETs is partly responsible for the poor prognosis after treatment, as the mechanisms through which NETs coat tumour cells and promote resistance to chemotherapy, immunotherapy and radiotherapy have already been described.^9^, ^52^, ^53^, ^54^ Under certain chemical conditions, tumour cells secrete interleukin‐1β (IL‐1β), which subsequently induces the generation of NETs. The formation of a NET necessitates the presence of two essential proteins: integrin‐αvβ1 and matrix metalloproteinase 9, which can trap and activate TGF‐β. Activation of TGF‐β instigates the EMT in cancer cells and is associated with the onset of chemoresistance.^55^ The resistance offered by NETs may be attributed to their structure, as NETs that develop in tumour tissues act as shields to prevent tumour cells from interacting with therapeutic drugs.^56^ Furthermore, the chemical composition of NETs could also be a key factor for resistance; for example, IL‐17, an IL discovered in NETs, could interact with cytotoxic CD8 T cells and exclude them from tumour tissues.^57^

## 4 THE CROSSTALK BETWEEN NEUTROPHILS AND OTHER CELLS IN TUMOUR MICROENVIRONMENT

### 4.1 Neutrophils and myeloid‐derived suppressor cells

**FIGURE 3:**
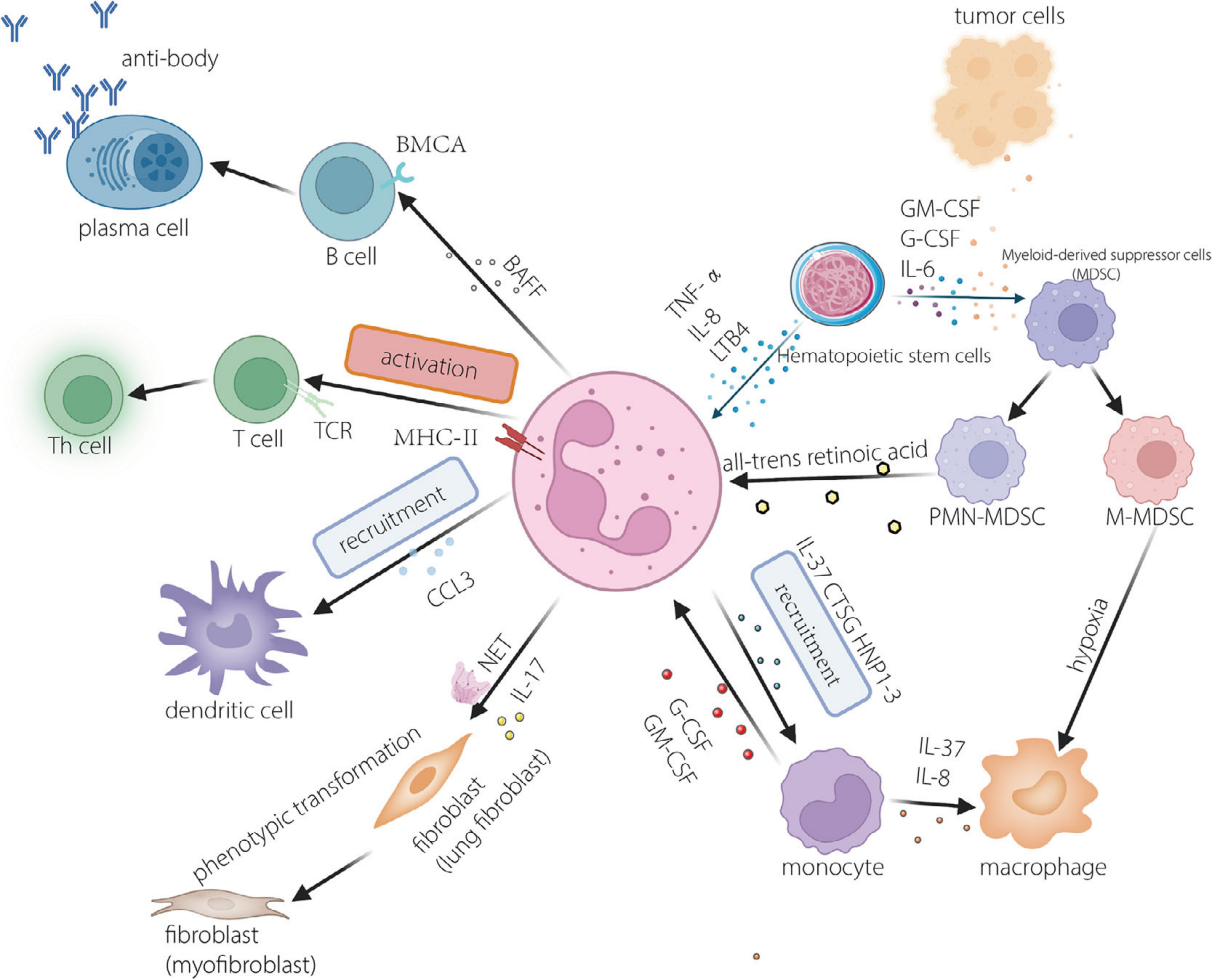
Crosstalk between neutrophils and other autologous cells. Myeloid‐derived suppressor cells (MDSCs), a certain type of immature granulocyte and neutrophil that originate from the same progenitors, result in diverse phenotypes because of the stimuli secreted by tumours. Monocytes can be recruited by cytokines released by neutrophils and differentiate into macrophages; in turn, granulocyte colony‐stimulating factor (G‐CSF) and GM‐CSF produced by monocytes can promote the germination of neutrophils. B cell receptor(BCR) and T cell receptor(TCR), which serve as a peculiar receptors, can be specifically recognised and subsequently develop into functional components. chemokine (C‐C motif) ligand 3(CCL3) can recruit dendritic cells to boost the immunisation. Neutrophil extracellular traps (NETs) additionally play a noteworthy role in fostering cellular communication, thereby aiding in the coordination of components within the tumour microenvironment. Phenotypic transformation of fibroblasts in the tumour microenvironment is conducted by NETs generated by mature neutrophils.

Myeloid‐derived suppressor cells (MDSCs) are recognised as pivotal orchestrators in immune therapy, playing a vital role in shaping the foundation of the TME.^58^ These cells are intricately linked to neutrophils and monocytes, manifesting exclusively in the context of cancer and other pathological conditions.^59^ Additionally, a study in the past few years reveled that neutrophils and MDSCs share an origin^6^, ^60^ (Figure 3). Mainstream views suggest that MDSCs are divided into two categories, polymorphonuclear myeloid‐derived suppressor cells (PMN‐MDSCs) and monocytic myeloid‐derived suppressor cells (M‐MDSCs).^60^ In tumour‐free mice, the counterparts of PMN‐MDSCs and M‐MDSCs are neutrophils and monocytes, respectively, exhibiting analogous phenotypic characteristics.^61^ Pathologically activated PMN‐MDSCs inhibit the function of lymphocytes and nature killer cells; for example, MDSCs can induce the formation of Tregs, which suppress the body's own immunity.^62^ PMN‐MDSCs can additionally facilitate the onset and metastasis of malignancies through non‐immunological mechanisms, such as modulating the expression of low‐density lipoprotein and its receptor lectin‐like OX‐LDL receptor 1(LOX‐1).^63^ Annihilating MDSCs enhance the immune capability of the body to eliminate cancer cells and improve the efficacy of immune therapeutic strategies.^58^ However, the mechanism underlying the immunosuppressive effect was unclear until recently, when Bianchi et al. elucidated the involvement of CXCL1 in eradicating T cells associated with pancreatic ductal adenocarcinoma.^9^ Unlike normal neutrophils, MDSC plays only a protumour role and are harmful to the functioning of the immune system. Additionally, MDSCs are considered to be immature cells in the human peripheral blood circulation.^65^ After being attracted to tumour tissues, M‐MDSCs can differentiate into tumour‐associated macrophages if HIF‐1α is upregulated in the TME.^66^ In regard to the other kinds of neutrophils, the conversion of PMN‐MDSCs to TANs can be blocked by all‐trans retinoic acid.^67^

### 4.2 Neutrophils and macrophages

Over the past two decades, remarkable strides have been achieved in the realm of tumour‐related research, and the concept of mutual recognition has been proposed that the process of tumourigenesis is led by immune dysfunction.^68^ Neutrophils and macrophages represent two pivotal constituents within the TME and play a role in every facet of tumour development and metastasis. Tumour‐associated macrophages and their precursor cells, monocytes, all interact with neutrophils. One study demonstrated that neutrophils can recruit monocytes by releasing IL‐37,^69^ and these recruited monocytes can develop into macrophages via the effects of IL‐8 and tumor necrosis factor(TNF)‐α^70^ (Figure 3). Additionally, a recent study on intrahepatic cholangiocarcinoma revealed that macrophages and neutrophils generate higher levels of oncostatin M and IL‐11, which both in turn activate that STAT3 signalling pathway and suppress the protumour function of macrophages and neutrophils.^71^ According to Tam's study, nucleotides released by macrophages exert a function as chemotactic factors facilitating neutrophil chemoattraction.^72^

To date, most studies on the interactions between neutrophils and macrophages have focused only on inflammation. Hence, it is imperative to conduct additional investigations to ascertain whether the interplay between neutrophils and macrophages during inflammation mirrors that observed in tumourous environments.

### 4.3 Neutrophils and other cells

Cancer cells possess the capacity to dynamically modify the TME by releasing an array of cytokines, chemokines and other factors. Consequently, this process can induce a reprogramming of adjacent cells, endowing them with the capability to play a central role in the advancement of cancer.^73^ In addition to fibroblasts, the microenvironment compromises immune cells such as macrophages, dendritic cells and adaptive immune cells, for instance, T and B lymphocytes. CCL3, a chemokine released by neutrophils, plays a major role in dendritic cell recruitment^74^ (Figure 3). Dendritic cells, often referred to as natural adjuvant,^75^ bridge the gap between the innate immune system and adaptative immune system. However, owing to their plasticity and the particular environment in tumours, dendritic cells always exhibit immune suppression ability. T lymphocytes in the TME secrete IL‐17, which influences stromal cells to release IL‐8 and granulocyte colony‐stimulating factor (G‐CSF) to recruit and boost neutrophils.^76^ In turn, the antimicrobial peptide cathelicidin generated by neutrophils is able to facilitate T‐lymphocyte differentiation and further protect them from apoptosis.^77^ Cancer‐associated fibroblast‐derived CLCF1 facilitates tumour progression by increasing the differentiation and infiltration of N2 neutrophils in the TME. As discussed above, N2 neutrophils are a specific subset of neutrophils that have a protumour function.^78^ Therefore, it can be concluded that cancer‐associated fibroblasts could enhance the progression.

## 5 PROGRESS IN NEUTROPHIL PHYSIOLOGY STUDIES

### 5.1 Ageing and neutrophils

Alterations in the immune system associated with ageing contribute to the heightened vulnerability of elderly individuals to infectious diseases, and potentially, autoimmune diseases and cancer.^79^ The different statuses of the human immune system may explain why patients of ages with different cancers have different susceptibilities. Bonaventura et al. reported that neutrophil ageing can alter neutrophil functions in relation to host age in his research on neutrophil biology.^80^ Decreased chemotactic responses to stimuli and the reduced phagocytosis are detected in neutrophils from elderly individuals.^81^, ^82^ From the present study, it is reasonable for us conclude that the alternations in neutrophil function are largely due to changes in signalling pathways, such as the MAP kinases, Jak/STAT and PI3K–Akt signalling pathways.^83^ Maurizio Sabbatini has explored into a rarely investigated field that the alternation of NET are attributed to ageing and drawn a conclusion that the elderly population have a greater amount of neutrophils but less stimulation of keratinocyte proliferation, which ultimately result in a poor prognosis in cancer patients.

### 5.2 Metabolism and neutrophils

Despite the differences in phenotypes and functions of TANs, all TANs still share one characteristic: to fulfill their functions within intricate tumour environments, these entities necessitate a profusion of energy and rapid metabolic adaptation.^84^, ^85^ Neutrophils can utilise many nutrients for life activities, including glycolysis, the pentose–phosphate pathway, fatty acid metabolism and glutamine metabolism.^86^ Several secreted factors can regulate the metabolism of neutrophils, among which G‐CSF could be a major functioning cytokine.^10^ G‐CSF not only increases glucose extraction and adenosine triphosphate(ATP) level^87^ but also promotes haematopoietic stem cell differentiation into neutrophils and the survival of neutrophils in circulating blood.^88^ The extracellular environment can also alter the metabolism of neutrophils, possibly through hypoxia. Hypoxia can diminish the generation of ROS, consequently resulting in an escalation of dysregulation induced by signalling mediators, all the while extending neutrophil survival by impeding apoptosis.^89^, ^90^

## 6 CANCER TARGETING THERAPY

The specific changes in neutrophil function are important for tumour initiation, metastasis and angiogenesis. Many clinical studies which aimed at targeting certain therapeutic sites have already been conducted (Table 1). Both the certain inhibition of neutrophil functions and a reduction in neutrophil numbers have led to few safe and efficacious therapeutic approaches.^17^, ^91^, ^92^, ^93^ These targets do not need to be unique to the neutrophil surface, and the functions of therapies could be direct or indirect. However, progress in targeting neutrophil therapy is being made.

**TABLE 1:** A brief conclusion of newly developed neutrophil‐targeting therapies.

Targets	Drug name	Cancer applications	Phase (identifier)	Alone or in combination	Mechanism	Ref.
CXCR2	Reparixin	Breast cancer	II (NCT01861054)	Alone	Reparixin, a probing allosteric inhibitor of CXCR2 within cells, has the capacity to diminish focal adhesion kinase(FAK) phosphorylation. This, in turn, mitigates the original phosphorylation of RAC‐aipha serine/threonine‐protein kinase(AKT) and the activation of the Wnt pathway responsible for governing the renewal of stem cells.	^94^
	AZD5069	Prostatic cancer	II (NCT03177187)	AZD5069 + enzalutamide	AZD5069 recognised by CXCR2 could improve the lack of the tumour suppressor phosatase and tensin homolog(PTEN), which can inhibit cancer cell survival through the inactivation of aldose reductase(AR), hypoxia induced factor‐1(HIF‐1) and nuclear factor‐kappa B(NF‐κB) transcription factors.	^95^
	AZD5069	Squamous cell carcinoma of head and neck	II (NCT02499328)	AZ9150 + MEDI4736 + AZD5069	The use of AZD9150 could weaken the nucleotide‐biding oligomerization domain containing 1/receptor interacting protein 2 (NOD1/RIP2) signalling pathway which could regulate proinflammatory cytokines IL‐8. In addition IL‐8 are found to stimulate cell proliferation in squamous cell carcinoma of head and neck.	^96^
CXCR4	68Ga‐pentixafor	Haematological malignancies	Not applicable (NCT05255926)	Alone	This treatment is designed to assist in determining the stage of the tumour and the efficacy of available therapies in relation to haematological malignancies as the recognition between cxcr4 and 68Ga‐pentixafor is representative in tumour areas.	^97^
	BMS‐936564	Acute myelogenous leukaemia	(I) NCT01120457	Alone	BMS‐936564, a pioneering IgG4 fully human monoclonal antibody, exhibits selective binding affinity to the second extracellular loop of CXCR4. This interaction, pivotal in the context of acute myelogenous leukaemia, instigates a substantial elevation, ultimately triggering apoptosis in tumour cells.	^98^
	BL‐8040	Pancreatic cancer	(II) NCT02907099	Alone	BL‐8040, a diminutive synthetic peptide, exhibits a robust affinity for CXCR4 (half‐maximal inhibitory concentration ∼1 nM). Its inhibitory effect on CXCR4 function precipitates a swift and enduring elevation in total white blood cell and lymphocyte counts.	^99^
	Plerixafor	Pancreatic, ovarian, colorectal cancer	(I) NCT02179970	Alone	Plerixafor disrupts the binding between the cancer cell‐associated CXCL12–KRT19 heterodimers and CXCR4 receptors on T cells. This interference facilitate the recruitment of T cells into the proximity of cancer cell clusters.	^100^
CD47	Hu5F9‐G4 (margolimab)	Acute myelogenous leukaemia	(I) NCT20678338I	Alone	Hu5F9‐G4 tightly associates with human CD47, obstructs the CD47–SIRPα interaction, amplifies phagocytic activity, and facilitates the engulfment of primary acute myelogenous leukaemia(AML) cells in vitro. It eradicates human AML in xenograft models and collaborates synergistically with rituximab to eliminate non‐hodgkin lymphoma(NHL) xenografts.	^101^
	ALX‐148	Recurrent platinum‐resistant ovarian cancer	(II) NCT05467670	ALX‐148 + doxorubicin + pembrolizumab	ALX‐148 specifically blocks the cd47 receptor, and since CD47/SIRPα axis additionally constrains the efficacy of tumour‐opsonising antibodies, a combination therapy pf ALX‐148 and other anticancer drug could boost their effect in platinum‐resistant ovarian cancer.	^102^
	TTI‐621	Solid tumours	(I) NCT02890368	Alone or with PD1 therapy	TTI‐621 (SIRPαFc) is a fully human recombinant fusion protein that also is recognised by CD47 and block the CD47–SIRPα axis and enhances phagocytosis of malignant cells.	^103^
GMR	GM‐CSF	Breast cancer	(II) NCT02636582	Alone	Tumour‐derived GM‐CSF functions as the pivotal regulator governing ARG1 expression in myeloid cells, contributing to local immune suppression. The blockade of granulocyte macrophage colony stimulating factor receptor(GMR) could significantly block the formation of an inhibitory tumour microenvironment.	^104^
PDE5	Tadalafil	Squamous cell carcinoma of head and neck	(I) NCT02544489	Alone	Inhibiting PDE leads to elevated cyclic guanosine monophosphate(cGMP) levels, causing the destabilisation of inducible nitric oxide synthase(iNOS) mRNA, diminished synthesis of iNOS, and ultimately a reduction in nitric oxide(NO) production. Taken together, these effects result in immunosuppressive properties of MDSCs and finally lead to the antitumour effect.	^105^
TRAILR2	DS‐8273a	Colorectal cancer	(I) NCT02991196	DS‐8273a + nivolumab	DS‐8273a, an agonistic antibody directed against the human DR5, demonstrates cytotoxic effects on human cancer cells and induces apoptosis through specific binding to DR5.	^106^

Abbreviations: CXCR, chemokine receptor; DR5, death receptor 5; IL‐8, interleukin‐8; MDSC, myeloid‐derived suppressor cell. TRAILR2: TNF‐related apoptosis inducing ligand receptor 2

Owing to the rapid development of biotechnology, several new targeting therapies have been developed.

### 6.1 Targeting metabolism

Neutrophils represent the most prevalent type of leukocytes found in circulating blood. Together with their rapid rate of daily production and function in systemic immunity, therapies targeting all neutrophils are challenging and increase the risk of infection with other diseases since innate immunity is inhibited. It is easy to conclude that targeting the specific characteristics of TANs could be a potential feasible therapy. Remarkably, directing attention towards glutamine metabolism emerges as a compelling therapeutic avenue, given the essential requirement for these amino acids by both tumour cells and TANs.^84^ A recent investigation utilised mouse models with subcutaneously injected 4T1 breast tumours, which were administered JHU083, a glutaminase inhibitor. This treatment resulted in a decrease in G‐CSF levels and MDSC mobilisation, concurrently promoting enhanced apoptosis within the tumour cells.^107^

### 6.2 Targeting NET

As mentioned above, NETs can promote tumour initiation and metastasis. Platelets ensnared via NETosis have the potential to obstruct the ingress of circulating tumour cells into the immune system, consequently thwarting metastasis, particularly in situations devoid of immune system involvement and the counteracting effects of blood flow shear forces.^108^ This process represents a newly discovered therapeutic target. A recent research also demonstrated that platelet in platelets could result in inhibition of metastasis.^109^

The complement system also plays a role in tumour treatment. C5a, an important component that promotes chemotaxis and inflammatory mediators, can trigger PMN‐MDSCs to favour growth and metastasis in a process dependent on the formation of NETs,^110^ and blocking C5a, C5aR1 is effective at minimising tumour metastasis.

NE constitutes a specific category of serine protease typically residing in the primary granules of neutrophils. Until recently, studies have indicated that NE secretion and NET formation can cooperate to affect tumour growth and metastasis.^111^ NE secreted by neutrophils is called ELANE and kills different types of tumour cells while sparing normal cells.^112^ However, because of the presence of many protease inhibitors in the TME, NE activity is suppressed, leading to protection of only tumour cells.^113^ After tremendous efforts, we found that the porcine‐derived ELANE homologue could perfectly overcome this barrier, which is more resistant to serine protease inhibition, that is, the other ELANE homologues because its catalytic function is well preserved.^112^

### 6.3 Targeting drug delivery system

As observed, neutrophils, the most prevalent white blood cells in the bloodstream, assume pivotal roles in acute inflammation and immune responses against cancer. Neutrophil recruitment and adhesion and tissue infiltration are key pathological changes in cancer. Utilising these chemotactic characteristics and copying the genetic material into a specifically targeted drug carrier is a promising strategy in cancer therapy.^114^ Two predominant categories of drug delivery systems exist: neutrophil carriers and nanovesicles derived from cellular membranes.^114^, ^115^

Recently developed chimeric antigen receptor (CAR) neutrophils have been innovatively engineered from pluripotent stem cells using CRISPR/Cas9‐mediated gene integration. This technique enables the expression of diverse, specific anti‐glioblastoma (GBM) CAR constructs. The primary objective of these engineered neutrophils is to efficiently traverse the blood–brain barrier and deploy nanodrugs responsive to the TME, thereby targeting GBM with precision. Loading CAR‐T cells with R‐Si*o*_2_‐TPZ nanoparticles, which can efficiently eliminate GBM cells, has been proven to be safe and effective in cancer therapy.^116^

Extracellular vesicles (EVs), which are generated by cells in vivo, are a natural prominent drug carrier systems due to their efficacy and cytotoxicity.^117^ They are membrane‐derived particles that work in intercellular recognition and communication. As neutrophils are recruited to cancer tissues, neutrophil‐derived EVs are also abundant. We then discuss how more EVs can be produced and what therapeutic agents can be loaded. Simpson reported that disruption of cultured cells by nitrogen cavitation could greatly increase EV production.^118^ In another study conducted by Coffelt et al., researchers combined neutrophil‐derived nanovesicles with NPs containing carfilzomib (CFZ), which is a second‐generation proteasome inhibitor. This complex is so called NM‐NP‐CFZ.^119^ According to previous studies, NM‐N0‐CFZ not only reduces the number CTCs, important tumour cells that function in tumour metastasis, in circulation, but also inhibits the formation of an early metastatic niche.

## 7 CONCLUSIONS

Above all, we have discussed the newly found relationship between tumours and neutrophils, and according to these findings, several auspicious therapeutic modalities have been devised to combat cancer. The significant impact of neutrophils in the TME has garnered widespread recognition. Despite the bright future of targeting neutrophils in cancer therapy, several questions, regarding cost, safety and reliability, still exist. For example, not only is the cost of targeting therapy much greater than that of normal chemotherapy, but the outcome is also questionable. Additionally, safety, which ranks first among all possible issue, is considered crucial because many patients may worry whether this targeting therapy will trigger other serious adverse reactions. The basic concern for ensuring the safety of targeting therapy is that the targets we utilise may be expressed on other cells and may be affected by targeted therapy, which could lead to dysfunction of normal tissues. Given that the targeting of neutrophils represents a novel domain in cancer therapy, numerous studies in murine models have been undertaken. However, the efficacy and safety of these agents for human application remain unverified. Because of technical limitations, many researchers have reported some uncertainties regarding the specific signalling pathways through which neutrophils endure cancer therapy, such as how this unique differentiation process works. Put differently, difficulties in progressing targeting neutrophil therapies are largely because research on different subsets of neutrophil is still in its infancy, as not all specific gene expression products have been identified and correctly analysing the data obtained from available high‐throughput sequencing technologies is a challenge.

In the coming years, there will be ongoing advancements in targeting neutrophil therapy through the rapid development of biological technology, shedding light on new breakthroughs in neutrophil investigations. Enhanced comprehension of the mechanisms underlying newly identified therapies can be substantiated through more intricate experiments, designed to more accurately replicate the complexities of the human physiology. The optimisation of targeting drugs should also be achieved by further study since targeted drugs are more costly than normal chemotherapy agents are, and the extent of drug application could be improved. A combination of targeted therapy and chemotherapy may be more valuable in clinical practice, as the side effects seem to be much lower with the available clinical studies. Researches on mechanisms and pathways associated with neutrophils could also contribute to the discovery of new biomarkers that could test whether existing therapies are useful for patients.

Taken together, these findings suggest that future cancer therapies could be promising for targeting neutrophils, but now, neutrophil‐targeting therapies are not the preferred option because of aforementioned concerns.

## AUTHOR CONTRIBUTIONS

Zhilei Bian, Weitang Yuan and Quanbo Zhou conceptualised the idea for the review. Drafts were written and revised by Shuaixi Yang and Jiachi Jia. All authors read and approved the final manuscript.

## CONFLICT OF INTEREST STATEMENT

The authors declare they have no conflicts of interest.

## ETHICS STATEMENT

Our review does not require ethical approval.
